# Supplementary material for: Mutation of GmIPK1 Gene Using CRISPR/Cas9 Reduced Phytic Acid Content in Soybean Seeds
Source: Int J Mol Sci. 2022 Sep 13;23(18):10583. doi: 10.3390/ijms231810583 (PMC9504718; doi:10.3390/ijms231810583)
Supplement: Supplementary file 1 [file ijms-23-10583-s001.zip › ijms-1874450-Supplementary materials.pdf]

Supplementary materials

IJMS, Song *et al.*

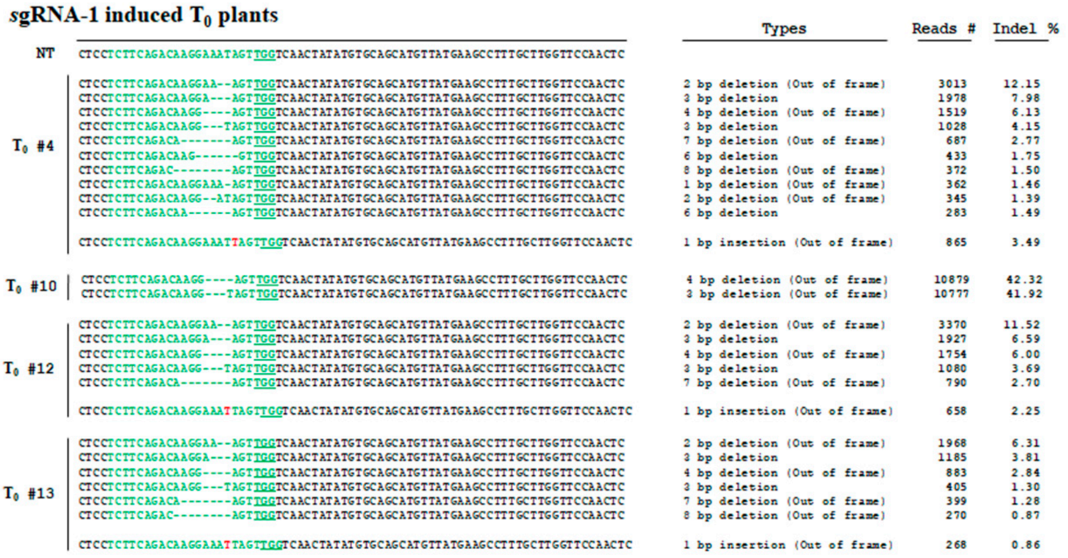

Figure S1. Inheritability of CRISPR/Cas9-induced mutations in *GmIPK1* gene-edited transgenic soybean plants (T_0) targeted by *GmIPK1* sgRNA-1. Four sgRNA-1 induced GE T_0 plants (Table S1) with high mutation ratios are presented. The mutation ratio (indel %) was calculated by dividing the number of reads containing indels at the target site (reads #) by the total number of sequencing reads. Protospacer adjacent motif (PAM) sequences (NGG) are shown in bold and underlined. Insertions and deletions are represented by the red and green hyphens, respectively.

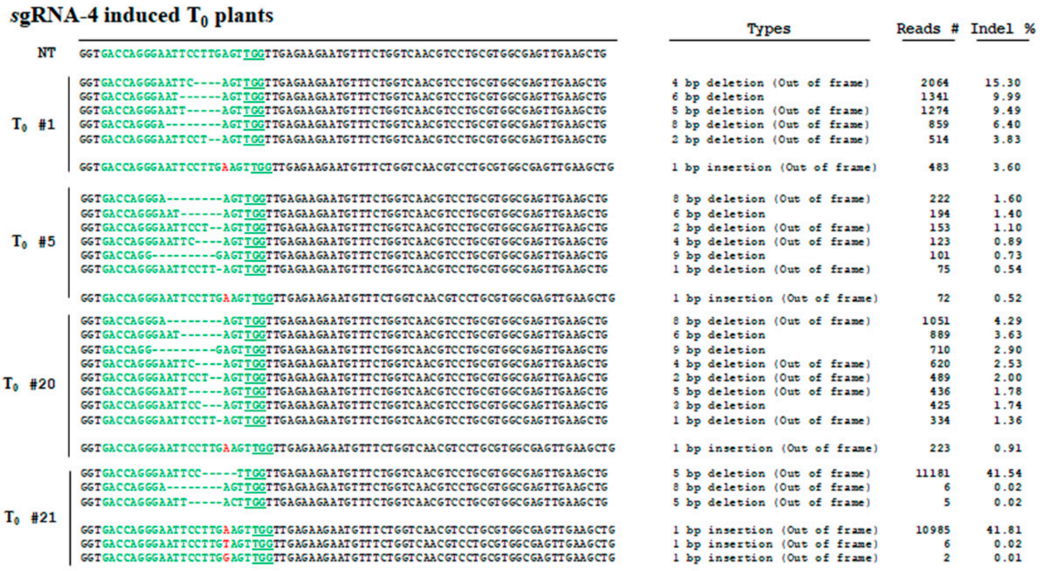

Figure S2. Inheritability of CRISPR/Cas9-induced mutations in *GmIPK1* gene-edited transgenic soybean plants (T_0) targeted by *GmIPK1* sgRNA-4. Four of sgRNA-4 induced GE T_0 plants (Table S2) with high mutation ratios are shown. The mutation ratio (Indel %) was calculated by dividing the number of reads containing indels at the target site (Reads #) by the number of total sequencing reads. The protospacer adjacent motif (PAM) sequences (NGG) are bold and underlined. Insertions and deletions are represented by red font and green hyphens, respectively.

Table S1. Indel (insertion and deletion) ratio (%) in *Gmipk1* sgRNA-1 induced T_0 plants

	Total reads	Insertions	Deletions	Indel frequencies (%)
NT	39145	16	696	712 (1.8%)
#2	21134	23	2010	2033 (9.6%)
#3	31317	0	44	44 (0.1%)
#4	24790	1021	15053	16074 (64.8%)
#5	25518	223	4293	4516 (17.7%)
#6	25290	0	2480	2480 (9.8%)
#7	30426	6	495	501 (1.6%)
#10	25709	6	21674	21680 (84.3%)
#12	29256	760	16093	16853 (57.6%)
#13	31124	323	8771	9094 (29.2%)

Table S2. Indel (insertion and deletion) ratio (%) in *Gmipk1* sgRNA-4 induced T_0 plants

	Total reads	Insertions	Deletions	Indel frequencies (%)
NT	18391	0	20	20 (0.1%)
#1	13429	620	9162	9782 (72.8%)
#2	27943	3	83	86 (0.3%)
#3	10128	19	289	308 (3.0%)
#5	14213	107	1712	1819 (12.8%)
#20	27268	376	9489	9865 (36.2%)
#21	26867	10974	11190	22164 (82.5%)

Table S3. Sequence analysis of mutations for the *GmIPK1* gene and its homologs in *Gmipk1* sgRNA-4 GE plants (T_2). The mismatched nucleotides between *GmIPK1* (*Glyma14g07880*) and the homologous genes (*Glyma06g03310* and *Glyma04g03240*) in *Gmipk1* sgRNA-4 GE plants (T_2) are marked in red, and the PAM sequences (NGG) are indicated in bold.

GE plants (T_1)	*Glyma14g07880* Indel frequencies (%)	Patterns	GE plants (T_2)	*Glyma14g07880* Indel frequencies (%)	*Glyma06g03310* Indel frequencies (%)	*Glyma04g03240* Indel frequencies (%)
NT	5 (0.0)		NT	77 (0.2)	0 (0.0)	0 (0.0)
#21-2	18503 (100.0)	5 bp deletion	#21-2-1	41977 (100.0)	0 (0.0)	0 (0.0)
			#21-2-2	46488 (99.9)	0 (0.0)	0 (0.0)
			#21-2-3	49816 (100.0)	0 (0.0)	0 (0.0)
			#21-2-4	48124 (99.9)	0 (0.0)	0 (0.0)
#21-3	24306 (100.0)	1 bp insertion	#21-3-1	49410 (99.7)	0 (0.0)	0 (0.0)
			#21-3-2	47478 (99.5)	0 (0.0)	0 (0.0)

Homologous genes	*GmIPK1* sgRNA-4 target sequence and its potential target sequences
Glyma14g07880 (GmIPK1)	GACCAGGGAATTCCTTGAGT**TGG**
Glyma06g03310	GCCAAGGGAATTTCTCGAAT**TGG**
Glyma04g03240	GTCAAGGGAATTTCTCGAAT**TGG**
